# Paracetamol and ibuprofen fixed-dose combination for the management of acute mild-to-moderate pain in children: strengthening and enhancing of result of Nominal Group Technique through Delphi consensus

**DOI:** 10.1186/s13052-024-01791-x

**Published:** 2024-10-29

**Authors:** Emanuele Castagno, Niccolò Parri, Antonio D’Avino, Elena Ferrari, Paola Giovanna Marchisio, Virginia Messia, Maurizio Taglialatela, Annamaria Staiano

**Affiliations:** 1 Department of Pediatric Emergency, Regina Margherita Children’s Hospital, Turin, Italy; 2 Department of Emergency Medicine and Trauma Center, Meyer Children’s Hospital IRCCS, Florence, Italy; 3 National Health Care Service, President of Federazione Italiana Medici Pediatri FIMP, Naples, Italy; 4 National Health Care Service, Federazione Italiana Medici Pediatri FIMP, Reggio Emilia, Italy; 5 Department of Pathophysiology and Transplantation, University of Milan, Milan, Italy; 6 Pediatric Unit, Fondazione IRCCS Ca’ Granda Ospedale Maggiore Policlinico, Milan, Italy; 7 Division of Rheumatology, ERN RITA Center, IRCCS Ospedale Pediatrico Bambino Gesù, Rome, Italy; 8 Division of Pharmacology, Department of Neuroscience, University of Naples Federico II, Naples, Italy; 9 Department of Translational Medical Sciences, Section of Pediatrics, University of Naples Federico II, Naples, Italy; 10 President of the Italian Society for Pediatrics (SIP), Rome, Italy

**Keywords:** Pain management, Pediatrics, Paracetamol/ibuprofen fixed-dose combination, Delphi consensus

## Abstract

**Background:**

Paracetamol and ibuprofen are the most commonly used drugs for pain treatment in children and their combination has shown improved analgesic effect compared to treatment with either drug alone. Current literature lacks specific guidelines regarding the settings in which this combination should be adopted.

**Methods:**

The survey, conducted with Delphi methodology, involved 75 hospital and outpatient pediatricians with clinical experience in the management of pain in children. Pediatricians involved were asked to validate or not the results of the previous NominalGroup Tecnique (NGT) consensus and thus specify the optimal clinical settings in which the paracetamol/ibuprofen fixed-dose combination could be adopted.

**Results:**

The results confirm the importance of the fixed-dose paracetamol and ibuprofen combination for the control of mild-to-moderate acute pain in children. Particularly, this association seems to be appropriate in case of headache, earache, odontalgia and musculoskeletal pain, and in specific settings such as post-operative and post-procedural pain. The broadening of the panel brought to slight variations in clinical management practices between hospital and outpatient specialists. Nonetheless, overall consensus supports the notion that the fixed dose combination is more efficacious than monotherapies and it is well tolerated. Moreover, experts unanimously agree on the usefulness of the combination for caregivers, leading to improved adherence and effectiveness.

**Conclusions:**

Both the NGT consensus and the broader Delphi consensus confirm the usefulness of the paracetamol-ibuprofen fixed-dose combination in pediatric pain. This is attributed to its superior effectiveness compared to monotherapies, a good tolerability profile, and improved compliance and ease of use. Some pain settings related to chronic, inflammatory and rheumatological pathologies remain to be investigated to evaluate the use of this combination.

## Introduction

Pain is common in children and requires special attention [1] as it can affect their physical and psychological well-being and cause stress for both patients and caregivers. Poor pain management during development is linked to short- and long-term effects on pain responses [2]. Yet, pediatric pain often remains underrecognized and undertreated [3, 4].

Paracetamol and ibuprofen represent the primary analgesics for managing acute traumatic and non-traumatic pain in children [5]. Current guidelines recommend their standalone or combined use for pain treatment [6, 7] and studies have assessed their concurrent administration for acute pain management in children [3, 8, 9].

The combination of paracetamol and ibuprofen provides better pain relief than using either drug alone [5, 10]. However, clear guidelines are needed for its use in different pediatric pain settings.

In 2022, an expert consensus, employing a Nominal Group Technique (NGT) process with a Board comprising 9 pediatricians and a pharmacologist, was conducted to elucidate the most appropriate use of the fixed-dose combination of paracetamol/ibuprofen in treating mild to moderate pain in children [11].

The NGT process highlighted the following key points [11]:

- paracetamol and ibuprofen fixed-dose combination is beneficial in postoperative pain management when both analgesic and anti-inflammatory effects are required, especially in cases where monotherapy with paracetamol proves ineffective;
- the fixed-dose combination can be used for postoperative pain in various settings, excluding the ENT (ear, nose, throat) region, where an elevated risk of bleeding due to non-steroidal anti-inflammatory drugs (NSAIDs) exists;
- the fixed-dose combination is particularly advantageous when monotherapy with either drug is ineffective, notably in cases of headache, earache, odontalgia and musculoskeletal pain;
- the complementary mechanism of action and the synergy between the two active molecules are critical for enhanced analgesic efficacy;
- the oral suspension facilitates optimized dosage based on the child’s weight, reducing dosing errors, and ensures faster absorption, leading to faster pain control.

Starting from the NGT process’ results, a Delphi survey involving a wider group of pediatricians and one pharmacologist with inpatient and outpatient expertise was designed to validate the previously obtained results and better specify the position in pediatric therapy for the newly introduced fixed-dose combination of paracetamol and ibuprofen.

## Methods

The investigation was conducted with a Delphi approach, an indirect, anonymous, iterative process aiming to achieve consensus among experts, particularly regarding disease management and drug therapy [12]. The study comprised 3 phases:

Exploratory phase:

- identification of the board members;
- project design and panel selection.

Analytical phase:

- literature update for the NGT process;
- revision of NGT conclusive statements;
- implementation of the Delphi survey.

Evaluation phase:

- a two-round Delphi survey;
- final data evaluation and discussion.

A Board composed by 7 pediatricians and 1 pharmacologist, was established, based on their experience in pediatric pain management.

Board participants had to satisfy the following selection criteria: documented clinical experience in pediatrics; being an active member of scientific societies dealing with children and pain management; having been involved in publications with pediatric pain as a topic. Notably, Board participants included members with different backgrounds and professional experience (e.g., general practitioner vs. hospitalist, size of the hospital, geographic location in the country).

Panelists were chosen from 400 Italian pediatricians who met at least three criteria: diverse professional backgrounds, relevant publications on the topic, active membership in pediatric societies dealing with pain management, or participation in national/international meetings. Of those invited, 74 (18.5%) voluntarily agreed to participate.

The Board analyzed the statements from the previous NGT consensus [11] to formulate a questionnaire for the Delphi survey.

The questionnaire, including 20 statements grouped in 5 different sections, was administered to panelists via a web platform that guaranteed anonymity.

Clinicians were asked to indicate their level of agreement on the proposed statements on a 5-point Likert scale (1: strongly disagree; 2: disagree; 3: neutral; 4: quite agree; 5: totally agree).

The consensus threshold was established at a minimum of 75% agreement from respondents who rated their level of agreement as ≥ 4 (either “quite agree” or “totally agree”). Statements falling below this percentage were deemed invalid.

In cases where the agreement rate ranged from 70 to 74% (indicating moderate agreement), or fell below 70% (indicating weak agreement), two additional indicators were considered for data interpretation: the mean score (categorized as strong: ≥4.2; moderate: 3.8–4.1; weak < 3.8) and the dispersion index (evaluating the heterogeneity of the ratings, deemed strong if < 45%).

The first questionnaire administered to the panel was sent to the 74 pediatricians, of whom 51 (68.9%) completed it. Data was analyzed and presented to the Board, both with a full table (including statistics for all statements) and with explanatory graphs for each statement. The board highlighted and discussed the statements whose outcome was unclear or inconsistent. Ambiguous or unclear sentences were edited or rephrased. Specifically, seven out of 20 statements were modified. Two statements were merged into a single question in the second questionnaire, bringing the final statements to 19.

The reviewed questionnaire was sent to the same 74 pediatricians of whom 52 (70.2%) completed it. Among these 52 physicians, 43 had also participated in the first consultation and 9 only in the second. Data analysis was presented to the board, with both a full table and explanatory graphs, and included a cross check between answers in the first and second rounds.

The study followed accurate methodology, ensuring a robust validation of expert consensus on the use of the fixed-dose combination of paracetamol and ibuprofen in pediatric pain management.

## Results

Overall, 60 pediatricians answered at least one of the two questionnaires, and the respondents were stratified by geographical area and by clinical activity. The percentage of hospital specialists was reduced between the first (41.2%) and the second round (32.7%), while the percentage of outpatient specialists increased (58.8% vs. 67.3%). **(**Table 1**)**

**Table 1:** Type of clinical activity and geographical distribution of participating panelists

Clinical Activity	First Consultation	Second Consultation
Mainly or exclusively in a hospital	41.2%	32.7%
Mainly or exclusively in outpatient services	58.8%	67.3%
**Geographic area**		
North-West (Piemonte, Valle d’Aosta, Lombardia, Liguria)	7.8%	17.3%
North-East (Trentino-Alto Adige, Veneto, Friuli Venezia Giulia, Emilia Romagna)	19.6%	17.3%
Center (Toscana, Umbria, Marche, Lazio)	23.5%	23.1%
South and Islands (Abruzzo, Molise, Campania, Puglia, Basilicata, Calabria, Sicilia, Sardegna)	49.0%	42.3%

Based on the agreement percentage, consensus was achieved for 15 out of 20 and 14 out of 19 items in the first and second round, respectively. For each statement the percentage of agreement and the mean score are reported. Statements 1–2 of section I; 5–7 of section III; and 4 of section V were rephrased/modified from the first round. Statements 1–2 of section II in the first round merged into a single statement in the second consultation (statement 1 of section II). The statements in the Table 2 refer to the questionnaire as presented to the participants in the second round.

**Table 2:** Responses to the survey

Statement*	First round (*N* = 51)		Second round (*N* = 52)	
	% with agreement ≥ 4	Mean score	% with agreement ≥ 4	Mean score
**I**. **I would use the fixed-dose combination**:				
1. In the post-operative period when the pain is not controlled after the administration of paracetamol or ibuprofen at an adequate dose**	74.5	3.9	88.5	4.3
2. In post-operative pain regardless of the type of operation when a greater analgesic effect than paracetamol alone is desired**	78.4	4.1	65.4	3.8
3. In post-operative pain when you want to obtain both an anti-inflammatory and analgesic effect	75.0	4.0	82.4	4.2
4. In mild-moderate post-procedural pain in case of ineffectiveness of first-line therapy	86.3	4.2	78.8	4.2
**II**. **I would use the fixed-dose combination**:				
1. In case of persistence of symptoms after the administration of paracetamol or ibuprofen at an adequate dose^†^	74.5	3.9	82.7	4.1
	64.7	3.7		
**III**. **The fixed-dose combination is effective in case of**:				
1. Headache	84.3	4.1	80.8	4.2
2. Dental pain	86.3	4.3	88.5	4.4
3. Earache	82.4	4.1	78.8	4.2
4. Musculoskeletal pain	80.4	4.0	73.1	3.9
5. Pain associated with chronic rheumatological diseases**	54.9	3.6	63.5	3.7
6. Pain associated with chronic inflammatory diseases**	62.7	3.7	59.6	3.7
7. Sore throat**	49.0	3.3	51.9	3.3
**IV**. **The oral suspension of the fixed-dose combination is to be preferred because**:				
1. It allows a simple and accurate definition of the dose based on body weight	86.3	4.3	82.7	4.2
2. Maximizes the correctness of the dosage in relation to the weight of the child	86.3	4.3	88.5	4.3
3. Reduces dosing errors by parents	76.5	4.2	78.8	4.2
**V**. **The advantages of using the fixed concentration combination are**:				
1. Effectiveness due to the complementarity of the mechanisms of action	86.3	4.3	93.3	4.3
2. Effectiveness due to the synergy between the effects of the two substances	94.1	4.4	78.8	4.1
3. Superior analgesia compared to single drugs used in monotherapy without compromising tolerability	86.3	4.2	80,8	4.1
4. The association of a component with a faster onset of analgesic action (paracetamol) with one with a longer duration of analgesic action (ibuprofen)**	72.5	4.0	75.0	4.1

*The statements presented refers to the questionnaire as presented to the participants in the second round

**Statements rephrased/modified from the first round

^†^Statement that derives from the integration of two different statements from the first round

The statement 2 of group I, concerning the use of the combination in post-operative pain regardless of the type of operation when a greater analgesic effect than paracetamol alone is desired, was validated in the first round (78.4%), but lost agreement in the second (65.4%) with an average score of 3.8 and a dispersion index of 56.7%. The agreement for using the combination in muscoloskeletal pain (statement 4 of section III) reached moderate agreement in the second consultation (73.1%) with intermediate scores for the mean score (3.9) and the dispersion index (47.4); however, it was considered validated in the first round (agreement ≥ 4, 80.4%). Statements 5–7 of section III, regarding the use of the combination in chronic diseases (inflammatory and rheumatological) and in sore throat, were not validated in the first round (agreement 54.9%, 62.7% and 49%, respectively) and, despite having been rephrased, did not reach agreement even in the second round (63.5%, 59.6% and 51.9 respectively).

## Discussion

The results of the previous NGT consensus established the potential usefulness of the fixed-dose combination of paracetamol and ibuprofen for the control of mild-to-moderate acute pain in children [11].

Overall, the above reported results achieved in a broader Delphi consensus reaffirmed the conclusions reached by the NGT Board. However, variations were observed on specific topics, that could be attributed to the heterogeneity of clinical practices among pediatricians with different backgrounds, experiences, type of clinical activity and geographical locations.

All statements will be briefly discussed with a major focus on the more controversial ones or those that have not achieved consensus.

In the first section, the panel reached consensus on the use of the fixed-dose combination in post-operative scenarios, especially when pain persists after paracetamol or ibuprofen when given at adequate doses. Agreement extended to its second line use for mild-to-moderate post-procedural pain, aligning with European [6] and American guidelines [7] endorsing the use of paracetamol and NSAID’s, either alone or in combination, in multimodal anesthesia protocols for post-operative and post-procedural pain. However, the statement advocating for the fixed-dose combination’s use in post-operative pain regardless of the operation type failed to reach consensus. The rewording, intentionally elusive about the type of intervention, likely contributed to resistance among primary care pediatricians. This lack of consensus aligns with the NGT Board’s position, which supported post-operative use, but excluded ENT and abdominal surgery.

Despite some disagreements, numerous studies establish the efficacy and tolerability of the paracetamol/ibuprofen combination in post-surgical pain, superior to monotherapies [3, 13] and offering a more favorable tolerability profile than opioids [14]. Concerns about bleeding risk in ENT surgery, associated mainly with NSAIDs, are not increased by the paracetamol combination [14].

The reluctance to endorse the combination may be influenced by well-established post-operative analgesic protocols in Italy that often involve drugs other than paracetamol and may not leave space for the paracetamol/ibuprofen association.

Additionally, guidelines recommend combination use only in case of monotherapy ineffectiveness [6, 7], a condition absent in the proposed statement. The panel, however, expressed consensus (almost 83%) regarding the use of combination in case of monotherapy ineffectiveness (section II).

In the third section, the panel endorsed the NGT consensus regarding the use of the combination for specific painful conditions such as headache, earache, and odontalgia [3, 13].

While musculoskeletal pain fell just below the consensus threshold (73%), obtaining consensus in the first round, disagreement prevailed in both rounds for chronic rheumatological and inflammatory diseases. Such hesitancy reflects uncertainties about the usefulness of fixed-dose combination in chronic pain, partly due to limited experience and its primary indication for acute pain.

Even the last statement of this section, related to the use of the combination for treating sore throat, did not find agreement among the panelists. This hesitation may have resulted from the concern that a symptomatic treatment might mask an infection requiring antibiotics.

This fear is heightened by the increased incidence of pharyngitis and streptococcal manifestations in the last two years [15], coupled with a lack of epidemiological data on the prevalence of rheumatogenic streptococcal strains, a crucial consideration for treating sore throat. Generally, guidelines recommend both paracetamol and ibuprofen for acute sore throat treatment, regardless of its etiology, which should be investigated [16]. Uncertainty in this area also arises from the heterogeneity of the pediatric population. While symptomatic treatment may be beneficial for infants not feeding due to pain, older children or adolescents may not require oral therapy at all, or perhaps a combination therapy.

Agreement was found for the statements in section IV, focusing on the use of the oral suspension, enabling more precise dosing based on the child’s weight, thereby reducing dosing errors by caregivers. Administration of multiple analgesics at home can be challenging, and fixed combinations improve patient adherence to therapy, enhancing analgesic efficacy [10, 14] and reducing dosing errors [17]. Liquid formulations are crucial for pediatric use and in conditions where swallowing is painful [10].

The last section (section V) addressed the advantages in terms of pharmacodynamics and pharmacokinetics of the fixed-dose combination of ibuprofen/paracetamol. The panel, persuaded by the complementarity and synergy of the two drugs, believes that the combination offers greater analgesic efficacy compared to monotherapies, maintaining a good tolerability profile [2, 14].

The rephrased last statement also gained agreement, emphasizing the unique properties of the two drugs, highlighting the faster onset of the analgesic effect of paracetamol and the longer duration of action of ibuprofen. Individual pharmacokinetics of paracetamol and ibuprofen remain unchanged with concomitant administration of a fixed-dose combination tablet, except for an increased rate of absorption of paracetamol, potentially accelerating the onset of the analgesic effect [17].

## Conclusions

The herein reported expansion of the previous NGT consensus using Delphi methodology reaffirms the importance of the fixed-dose paracetamol-ibuprofen combination in pediatric pain management.

The use for specific pain settings, such as odontalgia, earache, headache, post-operative and post-procedural pain, were identified, with some exceptions (e.g. ENT surgery). The study highlighted differences in responses based on the working setting of the respondents (primary care and hospitalist pediatricians), however, it overall confirmed the usefulness of paracetamol/ibuprofen fixed-dose combination therapy in pediatric pain management. This is attributed to its superior effectiveness compared to monotherapies, a good tolerability profile, and improved compliance and ease of use. Some areas, including musculoskeletal pain and pain caused by rheumatological and chronic inflammatory diseases, remain areas requiring further exploration.

## Data Availability

The authors confirm that the data supporting the findings are available within the article.

## Abbreviations

NGT: Nominal Group Technique
ENT: Ear, nose, throat
NSAID: Non-steroidal anti-inflammatory drugs
